# Overweight at age two years in a multi-ethnic cohort (ABCD study): the role of prenatal factors, birth outcomes and postnatal factors

**DOI:** 10.1186/1471-2458-11-611

**Published:** 2011-08-01

**Authors:** Marieke LA de Hoog, Manon van Eijsden, Karien Stronks, Reinoud JBJ Gemke, Tanja GM Vrijkotte

**Affiliations:** 1 Department of Public Health, Academic Medical Centre, University of Amsterdam, Amsterdam, the Netherlands; 2 Department of Epidemiology, Documentation and Health Promotion, Public Health Service of Amsterdam (GGD), Amsterdam, the Netherlands; 3 Department of Paediatrics, VU University Medical Centre, Amsterdam, the Netherlands

**Keywords:** body mass index (BMI), childhood, ethnicity, overweight, weight gain

## Abstract

**Background:**

Childhood overweight/obesity is a major public health problem worldwide which disproportionally affects specific ethnic groups. Little is known about whether such differences already exist at an early age and which factors contribute to these ethnic differences. Therefore, the present study assessed possible ethnic differences in overweight at age 2 years, and the potential explanatory role of prenatal factors, birth outcomes and postnatal factors.

**Methods:**

Data were derived from a multi-ethnic cohort in the Netherlands (the ABCD study). Weight and height data of 3,156 singleton infants at age 2 years were used. Five ethnic populations were distinguished: Dutch native (n = 1,718), African descent (n = 238), Turkish (n = 162), Moroccan (n = 245) and other non-Dutch (n = 793). Overweight status was defined by the International Obesity Task Force guidelines. The explanatory role of prenatal factors, birth outcomes and postnatal factors in ethnic disparities in overweight (including obesity) was assessed by logistic regression analysis.

**Results:**

Compared to the native Dutch (7.1%), prevalence of overweight was higher in the Turkish (19.8%) and Moroccan (16.7%) group, whereas the prevalence was not increased in the African descent (9.2%) and other non-Dutch (8.8%) group. Although maternal pre-pregnancy body mass index partly explained the ethnic differences, the odds ratio (OR) of being overweight remained higher in the Turkish (OR: 2.66; 95%CI: 1.56-4.53) and Moroccan (OR: 2.11; 95%CI: 1.31-3.38) groups after adjusting for prenatal factors. The remaining differences were largely accounted for by weight gain during the first 6 months of life (postnatal factor). Maternal height, birth weight and gender were independent predictors for overweight at age 2 years, but did not explain the ethnic differences.

**Conclusion:**

Turkish and Moroccan children in the Netherlands have 2- to 3-fold higher odds for being overweight at age 2 years, which is largely attributed to maternal pre-pregnancy BMI and weight gain during the first 6 months of life. Further study on the underlying factors of this early weight gain is required to tackle ethnic differences in overweight among these children.

## Background

Childhood obesity is a major public health issue [1,2]. In the Netherlands, trends over time show the prevalence of overweight and obesity in school-aged children increasing at an even a faster rate, and at earlier ages, than two decades ago [3,4]. However, an earlier study on childhood overweight showed a decline in overweight prevalence in Dutch girls (3-16 years) [5], and the obesity epidemic in European pre-school children seems to have stabilized [6].

Childhood overweight/obesity is directly related to a decrease in the quality of life of the child concerned [7], which emphasizes the importance of adequate prevention of overweight in children. Moreover, overweight/obese children are at increased risk of developing obesity at adult age which, in turn, is associated with various comorbidities, such as type II diabetes, cardiovascular disease, osteoporosis and depression [8-10].

Childhood overweight disproportionally affects some ethnic minority populations [11,12], which is consistently reported in American and European studies [5,13-16]. For example, Fredriks et al. found that overweight and obesity were much more prevalent in Turkish and Moroccan children compared to Dutch children, from age 2 years onwards [13].

Several factors can play a role in the development of overweight and, consequently, in ethnic disparities in overweight [17,18]. The developmental origins of disease hypothesis states that as early as the intrauterine period, environmental effects on human health and disease may occur as a result of prenatal, or even pre-conceptional characteristics of the mother, for example maternal body mass index (BMI), smoking, diabetes or hypertensive status [10,19-21]. Previous results from the ABCD study showed that pre-pregnancy BMI is an independent determinant for BMI of the child at the age of 14 months [22]. In the context of the child's BMI, however, controversy exists as to the associations and influences of prenatal factors, versus those of birth outcomes and early postnatal factors (e.g. early-life feeding conditions and rapid weight gain) [10,20,23,24]. In addition, the extent to which these risk factors for overweight account for ethnic differences in overweight is still unknown. Insight into these contributions is essential to develop effective strategies for early prevention of overweight, taking into account ethnic differences.

The present study explores ethnic disparities in overweight of children at age 2 years, and identifies and quantifies the prenatal factors, birth outcomes and postnatal factors that contribute to the explanation of these differences. We hypothesize that overweight at age 2 years will be more prevalent in children from ethnic minorities and that early life factors associated with overweight at early age may explain these differences.

## Methods

### Subjects

The Amsterdam Born Child and their Development (ABCD) study is a multi-ethnic birth cohort study conducted in Amsterdam, the Netherlands. The design and rationale of the ABCD study have been described previously [25]. In brief, between January 2003 and March 2004 8,266 pregnant women were included in the study after their first antenatal visit to an obstetric caregiver (phase 1). They filled out an extensive pregnancy questionnaire about socio-demographic data, obstetric history, lifestyle, dietary habits, and psychosocial conditions. Of these respondents, 7,863 women gave birth to a viable singleton infant and 6,575 women gave permission to collect information about their child's growth and infant feeding pattern, obtained from the Youth Health Care registration office of the public Health Service in Amsterdam (phase 2). Data on weight and height at age 2 years (104 weeks; range 100-160 weeks) were digitized for 3,432 children. The remaining children had moved to another city, or had missing data at age 2 years. Due to missing covariates the final sample for analysis comprised 3,156 children divided into five ethnic groups: Dutch native (n = 1,718), African descent (Surinam-Creole, Antilles, Ghana and other South Saharan African countries; n = 238), Turkish (n = 162), Moroccan (n = 245) and other (n = 793). The "other" group comprises children from different western and non-western countries. Ethnicity was based on the country of birth of the pregnant woman and her mother, to include both first-generation women (born outside the Netherlands) and second-generation women (born in the Netherlands, but whose mother was born in another country).

Ethical approval of the study was obtained from the Central Committee on Research involving Human Subjects in the Netherlands, the Medical Ethical Committees of participating hospitals, and from the Registration Committee of the Municipality of Amsterdam. All participating women gave written consent.

### Measurements

#### Main exposure

Height and weight were measured following a standard procedure by trained nurses, on average 14 times between birth and age 4 years. The primary outcome variable for this study was overweight (yes vs. no) of the child at age 2 years (mean age: 2 years and 3 months), which is a regular follow-up visit conducted at this time. Overweight was defined according to the gender-specific and age-specific International Obesity Task Force (IOTF) BMI guidelines [26]. BMI is a specific and moderately sensitive instrument designed to identify overweight in children from the age of 2 years onwards [26,27].

#### Potential explanatory factors

Three groups of potential explanatory factors related to childhood overweight were distinguished based on previous findings [10,18,28,29]: 1) prenatal factors, 2) birth outcomes, and 3) postnatal factors. Prenatal factors included maternal age (years), level of education (years of education after primary school: ≤5, 6-10, > 10 years), pre-pregnancy BMI (kg/m^2^) based on self-reported height and weight, maternal height (cm), hypertension (no, pre-existent, gestational), diabetes (no vs. pre-existent/gestational), parity (0, 1, ≥2), smoking habits (no, 1-5 or ≥ 6 cigarettes) and self-reported physical activity (no exercise, low, moderate, vigorous). Physical activity during the previous week was scored by calculating a Metabolic Equivalent (MET) score for the various reported activities using the compendium of physical activities [30]. Birth outcomes were birth weight (kg), gestational age (wk) and gender. Finally, postnatal factors were duration of exclusive breastfeeding (none, < 1 month, 1-3 months or > 3 months), and the average weight gain per month during the first 6 months of the child's life (g/mo). This was calculated as the difference between weight at age 6 months and the birth weight divided by the exact age at the 6-month examination.

### Non-response

The ethnic groups with growth data (response group n = 3156) were comparable to the ethnic groups (who gave birth to a viable singleton baby) without growth data (n = 4707) with respect to almost all measured demographic and lifestyle variables. However, Dutch mothers in the response group were slightly older (32.4 vs. 31.9 years) and higher educated (> 10 years after primary school: 59% vs. 53.9%). Multi-parity was more common in the Turkish mothers in the response group (61.3% vs. 51.5%), and the mothers in the "other" group were higher educated (> 10 years after primary school: 36.8% vs. 30.5%).

### Data analysis

Differences in baseline characteristics between ethnic groups were examined with χ^2^-tests (categorical data) or ANOVA (continuous data). After descriptive analysis of the study population, the association between overweight (including obesity), ethnicity, and the role of prenatal factors, birth outcomes and postnatal factors were assessed with multivariable logistic regression. Analyses followed a rational hierarchical format. First, univariable analyses were performed to determine the association of ethnicity and all other potential determinants separately with overweight. Subsequently, 3 models (forced entry) were built to assess the role of the potential explanatory factors in the association between overweight and ethnicity, which included only those covariates with a significance level of p < 0.10 in the univariable analysis in order to reduce the number of variables in the model. Correlations between the factors were all below 0.39, indicating no collinearity. In addition to ethnicity, model 1 included the prenatal factors: maternal age, height, pre-pregnancy BMI, education level, parity, diabetes and physical activity during pregnancy. Model 2 also included birth outcomes (birth weight and gender). The last model (model 3) included the postnatal factor: average infant weight gain during the first 6 months. Finally, we controlled for potential effect modification by use of interaction terms and we found no interactions between ethnicity and all potential explanatory factors in the univariable analyses (Wald test for interaction: p > 0.10), which means that the associations were the same across all ethnic groups.

The results are shown as odd ratios (ORs) and 95% confidence intervals (CI) using the ethnic Dutch group as reference. Statistical analyses were conducted using SPSS version 16.0 (for windows).

## Results

Table 1 presents the baseline characteristics according to each ethnic group. Dutch women were generally older and had a lower pre-pregnancy BMI compared to the other ethnic groups. The lowest BMI was found in the ethnic Dutch mothers (BMI: 22.6) and the highest in the Turkish (BMI: 24.6) and Moroccan (BMI: 25.1) mothers. Dutch infants showed less weight gain in the first 6 months and a lower BMI at age 2 years compared to the other ethnic groups.

**Table 1:** Characteristics of the study sample according to ethnic group

	Dutch N = 1718	African descent N = 238	Turkish N = 162	Moroccan N = 245	Others N = 793
**Prenatal pre-pregnancy factors**					
Age (year)	32.4 (4.1)^1^	29.0 (6.3)	26.5 (5.2)	27.6 (5.5)	31.3 (5.1)
Parity (%)					
*0*	60.9	46.2	39.5	47.3	54.5
*1*	31.7	27.3	35.8	27.3	32.4
*≥ 2*	7.5	26.5	24.7	25.3	13.1
Maternal education (%)					
*0-5 years*	7.0	42.6	56.5	54.2	22.4
*6-10 years*	34.3	47.8	38.5	40.8	40.0
*> 10 years*	58.7	9.6	5.0	5.0	37.6
Height (cm)	171 (6.2)	166 (6.2)	162 (5.1)	164 (6.7)	166 (7.2)
BMI mother (kg/m^2^)	22.6 (3.3)	24.4 (5.2)	24.6 (4.6)	25.1 (4.4)	22.7 (3.8)
Hypertension (%)					
Pre-existent	3.2	3.8	3.1	4.7	3.6
Gestational	11.8	8.9	5.0	7.2	7.4
Diabetes (%)					
Pre-existent	0.5	2.1	1.9	2.4	0.8
Gestational	0.1	0.8	0.6	0.0	0.4
Smoking (%)					
*None*	90.4	89.5	80.2	97.6	90.0
*1-5 cigarettes/day*	6.2	7.6	13.0	1.2	6.2
*≥ 6 cigarettes/day*	3.4	3.0	6.8	1.2	3.8
Physical activity (%)					
*Not*	6.6	34.5	32.7	34.7	20.1
*Low*	19.5	41.6	50.0	39.6	28.4
*Moderate*	32.0	16.4	11.7	20.8	23.8
*High*	41.9	7.6	5.6	4.9	27.7
**Birth outcomes**					
Birth weight (kg)	3.51 (0.55)	3.26 (0.62)	3.39 (0.53)	3.46 (0.50)	3.39 (0.54)
Gestational age (wk)	39.9 (1.7)	39.3 (2.0)	39.5 (1.8)	40.0 (1.5)	39.8 (1.7)
Gender (%)					
*Boy*	50.8	47.1	50.0	53.5	46.5
*Girl *	49.2	52.9	50.0	46.5	53.5
**Postnatal factors**					
Exclusive breastfeeding (%)					
*None*	18.5	18.9	5.6	12.2	18.3
*< 1 month*	7.1	12.6	17.3	13.1	8.6
*1-3 months*	30.7	40.3	31.5	42.0	30.9
*> 3 months*	43.7	28.2	45.7	32.7	42.2
Infant weight gain during the first 6 months (100 g/mo)	7.07 (1.34)	7.60 (1.56)	7.83 (1.50)	7.79 (1.47)	7.10 (1.35)
BMI 2 yr (kg/m^2^)	16.2 (1.3)	16.2 (1.4)	16.8 (1.7)	16.9 (1.7)	16.1 (1.4)
Overweight 2 yr (%)	7.1	9.2	19.8	16.7	8.8

^1 ^Mean (SD) (all such values)

Large differences in the prevalence of overweight in 2-year-old children among the ethnic groups were observed, with the highest prevalence among the Turkish (19.8%) and Moroccan (16.7%) children, compared with the Dutch children (7.1%).

Corresponding with the prevalence rates, the OR for overweight was increased in the Turkish (OR: 3.23; 95%CI: 2.11-4.96) and Moroccan (OR: 2.61; 95%CI: 1.77-3.84) group compared to the ethnic Dutch group. The odds for overweight in the African descent group (OR: 1.31; 95%CI: 0.81-2.13) and in the 'other' group (OR: 1.20; 95%CI: 0.88-1.64) did not differ from the ethnic Dutch group.

### Potential explanatory factors

The univariable analysis (Table 2) showed seven factors that were positively (+) or negatively (-) associated with overweight at age 2 years: maternal age (-), education level (-), maternal height (-), maternal pre-pregnancy BMI (+), physical activity level (-) (prenatal factors), birth weight (+) (birth outcome), and weight gain in the first 6 months of life (+) (postnatal factor). Parity, diabetes and gender were not associated with overweight at age 2 years; nevertheless, these factors reached significance level below p < 0.10 and were included in the multivariable analysis (Table 3).

**Table 2:** The odds ratio (OR 95% CI) of being overweight at age 2 years associated with selected prenatal factors, birth outcomes and postnatal factors

Overweight yes/no	N	OR (95% CI)
**Prenatal factors**		
Age mother (years)	3156	0.96 (0.94, 0.99)
Parity		
*0*	1768	1.00
*1*	991	1.28 (0.98, 1.67)
*≥ 2*	397	1.27 (0.93, 1.92)
Education		
*0 - 5*	614	1.76 (1.29, 2.41)
*6 -10*	1170	1.19 (0.89, 1.58)
*> 10*	1342	1.00
Height mother (cm)	3156	0.98 (0.96, 1.00)
BMI mother	3156	1.09 (1.07, 1.12)
Hypertension		
*None*	2717	1.00
*Pre-existent*	107	1.29 (0.70, 2.38)
*Gestational*	306	0.95 (0.62, 1.44)
Diabetes		
*None*	3120	1.00
*Pre-existent/gestational*	36	2.45 (1.06, 5.64)
Smoking		
*None*	2847	1.00
*1-5 cigarettes*	198	0.81 (0.56, 1.57)
*≥ 6 cigarettes*	109	1.19 (0.59, 2.12)
Physical activity		
*None*	492	1.00
*Low*	837	0.92 (0.64, 1.32)
*Moderate*	848	0.82 (0.57, 1.19)
*High*	978	0.63 (0.43, 0.91)
**Birth outcomes**		
Birth weight (kg)	3156	1.69 (1.34, 2.12)
Gestational age (weeks)	3156	1.00 (0.93, 1.07)
Gender child		
*Boy*	1566	1.00
*Girl *	1590	1.27 (0.99, 1.62)
**Postnatal factors**		
Excl. breastfeeding		
*None*	547	1.19 (0.85, 1.68)
*< 1 month*	280	1.40 (0.93, 2.13)
*1-3 months*	1022	1.06 (0.80, 1.42)
*≥ 4 months*	1307	1.00
Infant weight gain during the first 6 months (100 g/mo)	3156	1.58 (1.45, 1.72)

**Table 3:** Differences in prevalence of overweight at age 2 years associated with ethnicity using native Dutch children as reference (crude and multivariable adjusted Ors, 95% Cis)

Overweight yes/no	Crude	Model 1	Model 2	Model 3
	OR (95% CI)	OR (95% CI)	OR (95% CI)	OR (95% CI)
Ethnicity				
*Dutch*	1.00	1.00	1.00	1.00
*African descent*	1.31 (0.81, 2.13)	1.11 (0.65,1.92)	1.20 (0.69, 2.07)	0.79 (0.44, 1.41)
*Turkish*	3.23 (2.11, 4.96)	2.68 (1.58, 4.55)	2.64 (1.55, 4.51)	1.74 (0.99, 3.04)
*Moroccan*	2.61 (1.77, 3.84)	2.12 (1.33, 3.40)	2.07 (1.29, 3.33)	1.41 (0.86, 2.31)
*Others*	1.20 (0.88, 1.64)	1.21 (0.86, 1.70)	1.22 (0.87, 1.71)	1.14 (0.81, 1.62)

Abbreviations: OR = odds ratio; CI = confidence interval.

Model 1: including maternal age, height, pre-pregnancy BMI, education level, parity, diabetes and physical activity during pregnancy.

Model 2: as in model 1; with additional inclusion of birth weight and gender.

Model 3: as in model 2; with additional inclusion of infant weight gain during the first 6 months (100 g/mo).

### Prenatal

In the multivariable analyses, a decrease in ORs was found after adding the maternal prenatal factors in model 1. The highest contribution to the decrease in ORs in this model was due to the inclusion of maternal pre-pregnancy BMI (Table 3). On average, 17% of the ethnic differences in overweight were attributed to this single factor in the Turkish and Moroccan group. However, the OR for overweight remained higher in the Turkish (OR: 2.68; 95%CI: 1.58-4.55) and Moroccan (OR: 2.12; 95%CI: 1.33-3.40) groups compared to the ethnic Dutch group.

### Birth outcomes

Adding the birth outcomes in model 2 hardly attenuated the ORs in most ethnic groups (Table 3). The measured birth outcomes (birth weight and gender) were associated with overweight, but did not explain the ethnic differences.

### Postnatal

Finally, after adding the postnatal factor 'average infant weight gain during the first 6 months' in model 3, the largest decrease in ORs was seen (Table 3). On average, in the Turkish and Moroccan groups, 33% of the ethnic differences in overweight were attributed to early weight gain. The ORs for overweight became non-significant in the Turkish and Moroccan groups.

The final model (model 3), revealed that maternal pre-pregnancy BMI (kg/m^2^; OR: 1.07 95%CI: 1.04-1.10), maternal height (cm; OR:0.98; 95%CI: 0.96-1.00), birth weight (kg; OR:2.55; 95%CI: 1.95-3.34), gender (girls: OR:2.61; 95%CI: 1.96-3.48) and early weight gain (per 100 g/mo;OR:1.82; 95%CI: 1.65-2.01) were independently associated with overweight of the child at age 2 years.

## Discussion

This study examined ethnic differences in overweight in children of pre-school age, including the role of potential explanatory factors. Results showed that children from Turkish and Moroccan origin have a 2- to 3-fold higher OR for overweight at age 2 years compared to ethnic Dutch children. Children from African descent and those in the 'other' group did not have higher rates of overweight. Several factors associated with overweight were found, however, they did not necessarily explain the ethnic differences in overweight. Although the mechanisms of differences in childhood overweight in the Turkish and Moroccan infants are not completely understood, the explanatory role of maternal pre-pregnancy BMI, and mainly infant weight gain, were large.

Similar to our results, previous Dutch studies on ethnic differences in overweight found the largest prevalence of overweight (25-30%) in Turkish and Moroccan children [11,13]. Other US and European studies also found large ethnic differences in overweight [14,15,28,29,31]. The results of studies comparing overweight rates of African children with the natives are conflicting [10,15,16,28,31,32]. This absence of consensus may be due to different age groups and differences between the African countries included in these latter studies. In the present study, the 'African descent' group was based on comparable backgrounds and the children were relatively young. Most of the above-mentioned studies examined overweight prevalence in older children [5,14,29,31]. We showed that such differences in the Turkish and Moroccan children already exist at age 2 years. It is possible that overweight in African descent children becomes more prevalent at older ages.

Many risk factors for child overweight are more prevalent in ethnic minority groups [18]. As in our study, others have reported the importance of maternal pre-pregnancy BMI for ethnic differences in overweight in young children [10,22]. Recent results from the ABCD study, and a comparable study by Salsberry et al., showed that women from ethnic minority groups have the highest prevalence in overweight, with pre-pregnancy BMI as a strong predictor for high BMI of the pre-school aged child [10,22].

Several mechanisms could explain the association between pre-pregnancy BMI and weight/BMI of young children. 1) Genetic factors, which are passed from mother to child, could have a large effect on the variation in childhood BMI [33-35]. However, most studies on this topic have been conducted among Caucasian populations, while in populations with a lower socio-economic status, environmental effects may have a larger impact [36]. 2) Overweight in children may be propagated and enhanced at an early stage because of an abnormal metabolic environment *in utero*. Studies show that, in obese women, during gestation changes in hormonal mediators occur due to programming, that could result in more adverse adiposity metabolism in the child [20,21,37]. 3) A high BMI in the mother could result in enhanced foetal exposure to insulin and leptin, affecting its programming of energy balance and appetite regulation [21,37]. Finally, animal studies showed that overfeeding in early life (*in utero *and early postnatal) has a great impact on nutrient balance and hormone responses which can lead to a disturbance of appetite regulation [38].

This disturbance in appetite regulation can also influence the amount of weight gained in the first period of life, which we found to be the most important determinant explaining ethnic differences in overweight. Alternatively, early weight gain may be strongly related to social and/or environmental factors, particularly eating habits [23,24,39]. A long duration of breastfeeding (up to 4-12 months) is reported to be protective against childhood overweight [40,41] but was not confirmed in the present study; however, conflicting results have been reported related to breastfeeding. Other factors during the weaning period or shortly thereafter (e.g. establishing a family's diet and lifestyle) may overrule the effect of breastfeeding [41,42]. Alternatively, the young age of the participants in the present study might explain overweight. The ethnic differences in weight gain in the Turkish and Moroccan children may be due to cultural differences. Compared with ethnic Dutch parents, Turkish parents introduce fruit juices and snacks much earlier (6 vs 9 months) and both Turkish and Moroccan parents use supplemental feeding more frequently [43,44]. Furthermore, in Turkish families a 'chubby' infant is often perceived as a more healthy infant, and sometimes represents wealth and good motherhood [45]. The extra feeding and earlier introduction of solid food might promote overweight in early life in these children.

One strength of the present study is the large number of population-based participants, including many non-Dutch, followed from early pregnancy onwards. In addition, many known risk factors for overweight were included [17,46].

Nevertheless, some limitations need to be addressed. First, the Dutch mothers included in the present study (response group with growth data) have a higher educational level and were generally older. Due to this selection, the prevalence of overweight may be underestimated. Second, although this study is representative for the population living in the urban part of the Netherlands, some ethnic minority groups were slightly under-represented. Due to small numbers we were unable to analyse all ethnic groups separately. Therefore, interpreting the results (particularly for the 'other' group) was relatively difficult. Third the pre-pregnancy BMI of women was based on self-reported height and weight. Self-reported height tends to be slighted overestimated and weight underestimated, resulting in an underestimation of BMI [47]. Nevertheless, such underestimation will probably be very similar among all ethnic minority groups. Fourth, we had no data on paternal BMI and maternal weight gain during pregnancy. Paternal genetics and environmental effects are known to influence a child's growth [48]. Furthermore, maternal weight gain during pregnancy (specifically in combination with high pre-pregnancy BMI) is associated with birth weight and weight gain in the first period of life [49-51]. Finally, although odds ratios decreased (with an increasing number of explanatory factors in models), the confidence intervals reported in the multivariable analysis remained overlapping in the models. Therefore, these findings should be interpreted with caution.

## Conclusion

The high rate of overweight in the Turkish and Moroccan children, which already exists at age 2 years, emphasizes the need for better understanding of the explanatory factors contributing to this condition. The present study shows that maternal pre-pregnancy BMI and weight gain during the first 6 months of life made a large contribution to the ethnic differences in overweight. Future studies should focus on the underlying factors of early weight gain and the ethnic components, which may reveal new opportunities for the prevention of overweight in young children.

## Competing interests

The authors declare that they have no competing interests.

## Authors' contributions

MLAdH and TGMV developed the concept of the present study as part of the ABCD-study. MLAdH conducted the analyses. All co-authors provided statistical advice. MvE, RBJG and TGMV are the project managers of the ABCD study, and they co-ordinated the data collection. MLAdH was involved in obtaining part of the data. All the authors contributed to interpreting the results and writing the article, and they have seen and approved the final version

## Pre-publication history

The pre-publication history for this paper can be accessed here:

http://www.biomedcentral.com/1471-2458/11/611/prepub
